# The Role of Melanocortin Plasticity in Pain-Related Outcomes After Alcohol Exposure

**DOI:** 10.3389/fpsyt.2021.764720

**Published:** 2021-11-04

**Authors:** Nathan Sharfman, Nicholas W. Gilpin

**Affiliations:** ^1^Department of Physiology, School of Medicine, Louisiana State University Health Sciences Center, New Orleans, LA, United States; ^2^Neuroscience Center of Excellence, Louisiana State University Health Sciences Center, New Orleans, LA, United States; ^3^Alcohol and Drug Abuse Center of Excellence, School of Medicine, Louisiana State University Health Sciences Center, New Orleans, LA, United States; ^4^Southeast Louisiana VA Healthcare System (SLVHCS), New Orleans, LA, United States

**Keywords:** melanocortin, MC4R, pain, alcohol, opioids

## Abstract

The global COVID-19 pandemic has shone a light on the rates and dangers of alcohol misuse in adults and adolescents in the US and globally. Alcohol exposure during adolescence causes persistent molecular, cellular, and behavioral changes that increase the risk of alcohol use disorder (AUD) into adulthood. It is established that alcohol abuse in adulthood increases the likelihood of pain hypersensitivity and the genesis of chronic pain, and humans report drinking alcohol to relieve pain symptoms. However, the longitudinal effects of alcohol exposure on pain and the underlying CNS signaling that mediates it are understudied. Specific brain regions mediate pain effects, alcohol effects, and pain-alcohol interactions, and neural signaling in those brain regions is modulated by neuropeptides. The CNS melanocortin system is sensitive to alcohol and modulates pain sensitivity, but this system is understudied in the context of pain-alcohol interactions. In this review, we focus on the role of melanocortin signaling in brain regions sensitive to alcohol and pain, in particular the amygdala. We also discuss interactions of melanocortins with other peptide systems, including the opioid system, as potential mediators of pain-alcohol interactions. Therapeutic strategies that target the melanocortin system may mitigate the negative consequences of alcohol misuse during adolescence and/or adulthood, including effects on pain-related outcomes.

## Introduction

Alcohol use is estimated to be the third leading preventable cause of death in the United States (1). This estimate is likely outdated, as recent data indicates there has been an increase in alcohol-related deaths (2). Furthermore, with the recent COVID-19 pandemic, emerging data indicates escalated alcohol consumption globally that includes higher drinking in individuals with an alcohol use disorder, and also in individuals who were abstinent prior to the pandemic (3). Chronic alcohol use is associated with the development of pain disorders (4). Low to moderate alcohol drinking is associated with pain relief, and heavy alcohol use is associated with greater pain-like states (5). A large cross-sectional study from Brazil of nearly 2,300 adults revealed that excessive alcohol drinking was associated with an increased likelihood of chronic pain, whereas moderate alcohol consumption showed a somewhat protective effect against the incidence of chronic pain (6). Other studies have indicated similar findings, where individuals show hypersensitivity to pain-like (hyperalgesia) and innocuous (allodynia) stimuli following chronic alcohol consumption (5, 7).

These effects of alcohol consumption on pain sensitivity are not limited to adults, and there is evidence that similar hypersensitivities and development of pain states may occur in adolescents that use alcohol. For instance, in adolescents seeking treatment for chemical dependency, those who use alcohol are more likely to experience abdominal pains, headaches, and females are more likely to have painful reproductive-related issues such as endometriosis, than those who do not use alcohol (8). In college drinkers, hypersensitivity is seen in binge drinkers who have recently drank alcohol within the past 2 days relative to those who drank moderately and also within the past 2 days, or abstain from alcohol (9). The use of alcohol during adolescence has long-lasting detrimental effects on behavior and neurobiology (10, 11). Initiation of alcohol during the adolescent period predisposes individuals to life-long complications including increased likelihood of alcohol use disorder (AUD) and the development of psychological pathologies (12, 13). For instance, adolescents who drink before the age of 15 are four times more likely to develop alcohol dependence compared to those who initiate alcohol later in life (13).

Furthermore, initiation of alcohol as adolescents increases the likelihood of developing psychiatric disorders including depression, which may drive alcohol use in adulthood (14). Changes to underlying neurobiology are likely related to the behavioral outcomes in adulthood (4, 10, 15), thus it is necessary to elucidate potential systems that (1) are sensitive to alcohol, (2) modulate alcohol-related pathologies, and (3) can be targeted by conventional methods to alleviate alcohol-specific pathologies.

Alcohol use alters responses to innocuous and noxious stimuli in adolescents (8, 9) and adults (7, 16, 17) as well as neural processing of and behaviors to noxious stimuli (nociception) (18–21) and innocuous stimuli (allodynia) (22, 23) in preclinical models. Alterations in neuropeptide and neurotransmitter systems have been implicated in alcohol-induced alterations to behavior including corticotropin releasing factor (CRF), neuropeptide Y, calcitonin gene related peptide, and opioids to name a few (4, 15). In addition, alterations to such systems occur in brain regions implicated in both the progression of AUD and processing of pain information. Cortical and subcortical regions implicated in both pain processing and alcohol use include prefrontal cortical regions, the striatum, cingulate, insula, amygdala, and periaqueductal gray (4, 15, 24). Currently it is thought that enhancement of pro-stress neuropeptide systems underlies the transition from alcohol use to misuse (4, 24) and maladaptive behavioral outcomes including alcohol-induced pain sensitivity (19). However, one less examined neuropeptide system that is implicated in both alcohol-related outcomes and pain processing is the melanocortin system. We intend through this review to lay down a framework that supports the hypothesis that melanocortin system activity is associated with pain and alcohol use and enhances maladaptive outcomes such as alcohol-induced hyperalgesia. This effort is timely as negative outcomes following the pandemic will include increased substance use and side effects such as hypersensitivity, and identifying targets to mitigate both alcohol and pain can subserve a multitude of individuals.

## Overview of the Melanocortin System

**Figure 1:**
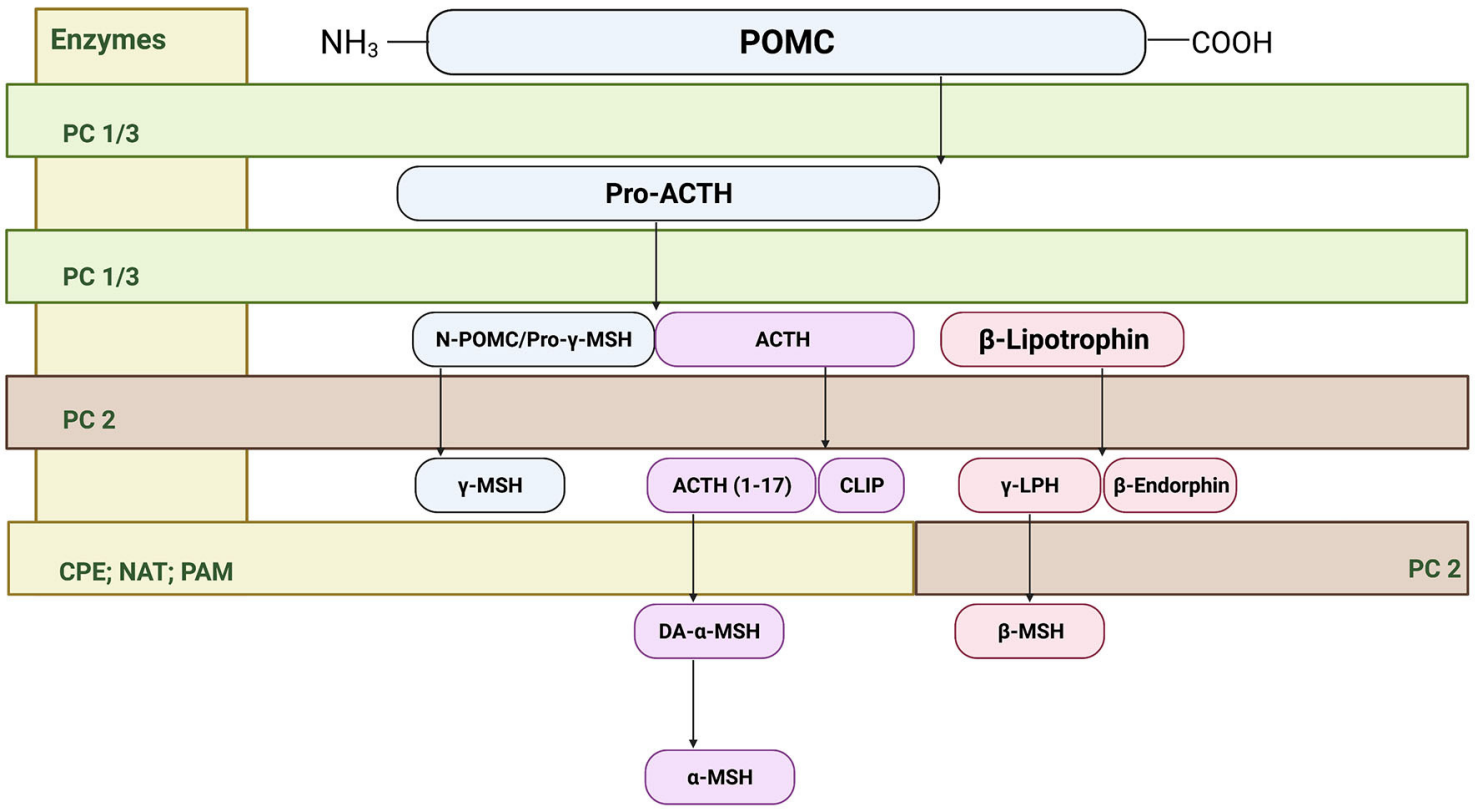
Cleavage of precursor protein pro-opiomelanocortin (POMC). Pro-hormone convertase 1/3 (PC1/3) cleaves POMC into pro-adrenocorticotropic hormone (Pro-ACTH) and β-lipotropin. PC 1/3 further processes pro-ACTH into ACTH where proconvertase 2 (PC 2) then sequentially cleaves ACTH to ACTH (1–17) and corticotropin-like intermediate peptide (CLIP). From there, carboxypeptidase E cleaves the basic amino acid residues of ACTH (1–17) allowing amidation by peptidyl α-ami-dating monooxygenase (PAM) to form des-acetyl-α-MSH (DA-α-MSH), where then n-acetyltransferase (NAT) acetylates DA-α-MSH into the mature α-MSH. PC2 also cleaves the N-terminal portion of POMC fragement/pro-γ-melanocyte stimulating hormone (MSH) into γ-MSH. Finally, β-lipotropin is processed into the endogenous opioid β-endorphin and γ-lipotrophin.

The melanocortin system was first described in the nervous system in late 1970's and 1980s (25), however it was not until the 1990's that the receptors for the melanocortin system were cloned (26, 27). Within the central nervous system, two predominant melanocortin receptors have been identified. The melanocortin 3 receptor (MC3R) has been primarily identified within regions of the hypothalamus, and in extra-hypothalamic regions in smaller amounts. In contrast, the melanocortin 4 receptor (MC4R) is widely distributed across the central nervous system (26–28). At the cornerstone of the melanocortin system is the prohormone pro-opiomelanocortin (POMC) that undergoes posttranslational processing by the enzymes proconvertase 1 (PC1), 2 (PC2), 3 (PC3), carboxypeptidase E, peptidyl α-ami-dating monooxygenase, and *n*-acetyltransferase. In the anterior pituitary, corticotrophs express PC1 and PC3 that post-translationally cleaves POMC into ACTH, β-lipotropin, and the N-terminal POMC fragment (29, 30). In the intermediate pituitary and hypothalamus, PC2 cleaves ACTH into ACTH 1–17 and corticotropin-like intermediate lobe peptide (CLIP). Furthermore, PC2 cleaves β-lipotropin into the opioid agonist β-endorphin and γ-lipotropin, which may be further processed into β-melanocyte stimulating hormone (31). Carboxypeptidase E, peptidyl α-ami-dating monooxygenase, and *n*-acetyltransferase generate the mature form of α-MSH from ACTH 1–17 (31) (see Figure 1). Importantly, these enzymes including PC1, PC2, and carboxypeptidase E are expressed in extra-hypothalamic brain regions, including the amygdala, hippocampus, cortex and ventral tegmental area (32–35). In addition to endogenous agonists, the melanocortin system is unique in that it also produces an endogenous antagonist agouti-related peptide (AgRP) (29). MC4R is a G-protein-coupled receptor that is coupled to the cAMP signaling pathways, where binding of α-MSH increases cAMP production in a dose-dependent manner (27, 36, 37); however, recent evidence also indicates that MC4R may signal through alternative pathways (38), which is explored in detail below.

The melanocortin system mediates a wide variety of behaviors and functions from grooming (39), sexual behaviors (27), cardiovascular tone (40), modulation of inflammation (41), to energy balance and feeding behaviors (29, 42, 43). Early evidence also indicated that the melanocortin system interacts with the opiate system in modulating pain transmission (44–46), and subsequent data showed that antagonism of the MC4R modulates tolerance to opioid effects on nociception (47, 48). Since this discovery, MC4R signaling in spinal and supra-spinal regions has been implicated in modulating emotional and sensory aspects of pain (49). Furthermore, MC4R signaling modulates alcohol consumption (50) and is sensitive to the effects of chronic alcohol exposure during the adult (51) and adolescent periods (52). Drug addiction including alcohol abuse can be conceptualized as a cycle of binging/intoxication, withdrawal/negative affect, and preoccupation/anticipation, whereby repeated usage of a substance places greater allostatic (maintenance of stability outside the homeostatic range) load further promoting substance use (15). Heavy use and/or binging of alcohol increases in pro-stress signaling in the brain, a key component underlying the neurobiology of withdrawal/negative affect stage of addiction, promoting further and escalated alcohol use (53, 54). For instance, corticotropin releasing factor (CRF) is implicated in driving stress adaptations of the hypothalamic-pitutitary-axis and brain stress systems (55); however, there is evidence suggesting that MC4R is a part of the physiological response to stress (56–58). Stress increases melanocortin tone and broad MC4R activation elicits anxiogenic and stress-like responses (58, 59). During the withdrawal/negative affective stage of addiction, it is therefore likely that there is enhanced melanocortin tone that contributes to substance abuse. In the following sections, we will summarize the state of the science on the relationship between MC4R signaling and alcohol use, alcohol misuse and pain.

## Melanocortin System and Pain

### Melanocortin and Pain Interactions

There is a growing body of evidence implicating MC4R signaling in modulation of pain-specific behaviors in multiple models that include nerve injury, alcohol- and opioid-induced pain, and inflammation (see Table 1). Early work implicated the melanocortin system in pain-related behaviors where injections with ACTH, which is converted into fragments that agonize melanocortin receptors (α-MSH), produced hyperalgesic responses in rabbits (78) and rats (60). Importantly, these studies laid the foundation for a functional interaction between the opioid system and the then, undiscovered, melanocortin system. For example, β-endorphin-mediated analgesia could be abolished by pre-treatment with α-MSH or ACTH in models of thermal nociception (78, 79) and electrical footshock (80). Furthermore, these effects were reliably elicited in adrenalectomized animals (60, 80) indicating that pain-like nocifensive (behaviors due to noxious stimuli) responses were not dependent on corticosterone. As the MC2R mediates ACTH-induced effects on hypothalamic-pituitary-axis engagement in response to a stressor (81), behavioral effects in adrenolectomized animals implicated other melanocortin receptor subtypes in the antagonism of opioid-induced analgesia. As ACTH has affinity for both MC4R and MC3R, with a slightly higher affinity for MC3R (693 vs. 86.9 K_i_ [nmol/L], respectively) (82), studies that utilize ACTH cannot rule out the possibility of effects on MC3R. However, as previously stated, most MC3R expression is limited to the hypothalamus, septum, ventral tegmental area, and raphe nucleus (46, 83), nonetheless alternative studies utilizing more specific agonists and antagonists of the MC4R system were needed.

**Table 1:** Melanocortin system interactions on pain-related outcomes.

**References**	**Pain condition**	**Animal model**	**Age and/or Weight**	**Drugs used**	**Main findings**
Sandman and Kaastin (60)	None	Sprague-Dawley rats	90 days	ICV administration •α-MSH (0.1, 1, 10 μg)	•ICV administration of α-MSH lead to hyperalgesia in normal rats
Beltramo et al. (61)	CCI	Male Sprague-Dawley rats	150-175g	None	•CCI Increased MC4R and precursor ligand POMC gene expression in spinal cord •Increased expression was generalized to spinal cord and not DRG
Bellasio et al. (62)	Formalin	Male CD1 mouse	25-30g	Intrathecal and/or ICV •MTII (5 nmol/mouse) •SHU9119 (5 nmol/mouse) •HS014 (5 nmol/mouse) •JKC-363 (5 nmol/mouse) •AgRP (1.5 nmol/mouse)	•MTII (I.t) decreased nociceptive thresholds in both phases of the formalin pain test •MC4R antagonists (SHU 9119; HS014; I.t. and ICV) lead to a dose-dependent reduction in hyperalgesic behaviors in second phase of formalin test
Ercil et al. (63)	Mouse model of obesity (A^y^ mice)	Male mice (C57Bl/6 background) •a/a—recessive •A^y^/a—dominant AgRP allele	8–10 weeks old	ICV •HS014 (0.0032, 0.032, 1 nmol) IP •Morphine (3.2, 10, 32 mg/kg)	Locomotor behavior •HS014 significantly shifted the inverse U-curve downward and largest dose (0.032 nmol) significantly decreased motor activity at baseline Antinociceptive behavior •A^y^ mice had significantly higher baselines and were. More sensitive to effects of morphine than C57Bl/6 mice •HS014 shifted the morphine dose-response curve three-fold to the left and increased baseline latencies at all doses tested indicating increased potency of morphine (only tested in C57Bl/6 mice)
Zvejniece et al. (64)	Formalin test Tail Flick (no pain condition)	Male ICR mice	23–25 g	Peripheral admin (subcutaneous): •α-MSH (0.1, 1 μmol/kg) •HS014 (0.1, 1 μmol/kg)	•During the formalin test α-MSH showed analgesic actions •During tail flick only HS014 showed analgesic actions similar to indomethacin
Vrinten et al. (65)	CCI	Male Wistar Rats	200–240 g	Intrathecal Admin: •SHU9119 (0.14, 0.46, 1.4 nmol) •MTII (14.6, 29.2, 97.6, 488 pmol) •D-Tyr-MTII (0.29, 0.96, 2.88 nmol) •Nle-γ-MSH (3.22 nmol) •Combination of MTII (14.6 pmol) and SHU919 (0.46 nmol)	•SHU9119 dose-dependently increased cold and mechanical thresholds in CCI, but not control rats •MTII and D-Tyr-MTII dose-dependently decreased cold and mechanical thresholds in CCI, but not control rats
Vrinten et al. (66)	CCI	Male Wistar rats	250–300 g	•SHU9119 (0.5-1.5 μg) •MT-II, (0.5 μg) •Morphine (1–30 μg) •Naloxone (0.1–100 μg) Combination •Naloxone (0.1 μg) + SHU9119 (1.5 μg) •Morphine (1–30 μg) + SHU9119 (0.5 μg) •MTII (1.5 μg) + Morphine (100 μg)	•SHU9119 decreased allodynia by CCI and pretreatment with subtherapeutic naloxone decreased SHU9119-mediated effects •Morphine and SHU9119 had an additive effect on anti-allodynia
Starowicz et al. (67)	CCI	Male Wistar rats	250-300g	Intrathecal Admin •SHU9119 (0.15, 0.5, and 1.5 μg) •MT-II, (0.03, 0.1, and 0.5 μg) •DAMGO (0.1, 0.25, and 0.5 μg) •Morphine (10, 20, and 30 μg) •Cyprodime (30 μg)	•SHU9119 highest does reversed CCI effects on thermal and mechanical hypersensitivity, only the other doses had effects on mechanical but not thermal hypersensitivity •MTII affected both injured and uninjured paw responses to thermal and mechanical stimuli. Dose-dependently increased sensitivity to thermal and mechanical stimuli •Cyprodime enhanced efficacy of SHU9119 and MTII •DAMGO increased mechanical thersholds that was decreased by either SHU9119 or MTII
Starowicz et al. (47)	Morphine tolerance	Male Wistar rats	200–350 g	Intra-Central Amygdala Injections •SHU9119 (0.15 or 1.5 μg) •αh-CRF (0.5 or 1 μg)	•Morphine acutely decreased MC4R mRNA and chronically increased MC4R mRNA •SHU9119 reversed morphine tolerance and enhanced morphine's antihyperalgesic and allodynic effect. 0.15 μg of SHU9119 enhanced thermal but not mechanical thresholds, whereas 1.5 μg enhanced both
Starowicz et al. (48)	CCI 7 days following they were treated	Wistar Rats	220–250 g	Intrathecal administration of: •SHU9119 (0.15 or 5 μg) •MT II (30 or 100 ng)	•Administration of SHU9119 resulted in dose-dependent increases in anti-allodynia that was diminished on day 14 •MT II heightened allodynic effect that was also decreased on day 14
Starowicz et al. (68)	Morphine tolerance (10 mg/kg i.p.)	Male Wistar rats	200–250	Intrathecal •SHU9119 (0.5 or 1.5 μg) •JKC-363 (0.7 or 2 μg)	•SHU9119 (1.5 μg) and JKC-363 (0.7 μg) prevented tolerance development of morphine, the lower dose had no effect •Single dose of drugs at both concentrations in combination with morphine were able to reinstate morphine efficacy on tail-flick; drugs alone had no effect on tail flick assay
Starowicz et al. (69)	CCI	Male Wistar rats	220–250 g	Intraplanar injection: •SHU9119 (2.8 or 11.2 nmol) •JKC-363 (4.0, 8.0 nmol)	•SHU9119 and JKC-363 reversed allodynia and hyperalgesia in CCI, effects were maximal between 15 and 30 min. •JKC-363 had longer effect than SHU9119
Kalange et al. (70)	Morphine withdrawal hyperalgesia	Male Sprague-Dawley rats	220-260g	Acute treatments: ICV administration •Morphine (2–20 μg/rat) •HS014 (0.008- 0.08 ng/rat) •NDP-MSH (0.04–0.12 ng/rat) Chronic treatmentICV administration •HS014 (0.008ng/rat) Osmotic Minipump •Morphine (20ng/μl/h)	In acute administration: •Morphine does dependently increased tail withdrawal (2 μg/rat was subthreshold) •HS014 dose dependently increased tail withdrawal (0.008 ng/rat was subtherapeutic) •NDP-MSH does dependently decreased tail withdrawal (0.04 ng/rat was subtherapeutic) Acute Combination of Morphine and: •NDP-MSH (0.04 ng/rat) antagonized morphines antinociceptive effect •HS014 (0.008 ng/rat) additively enhanced morphine analgesic effect Chronic Treatment with HS014 •Subtherapeutic HS014 prolonged morphine tolerance and prevented morphine-induced hyperalgesia Morphine-induced hyperalgesia •HS014 dose dependently reversed hyperalgesia
Chu et al. (71)	CCI	Male Wistar rats	280–320 g	Intrathecal admin for 7 days •HS014 (5 μg/day) •SB203580 (10 mg/day)	•Administration of HS014 resulted in anti-allodynia that was preserved after cessation of drug •Following CCI surgery both the group that received HS014 and the group that received SB203580 showed reduced signs of thermal and mechanical hypersensitivity relative to group treated with saline
Chu et al. (72)	CCI	Male Wistar Rats	200–250 g	PAG injections •HS014 (dose 1; 0.1 μmol/0.5 μL, dose 2; 1 μmol/0.5 μL)	•HS014 reduced thermal and mechanical hypersensitivity from CCI
Roltsch-Hellard et al. (20)	Alcohol-induced hyperalgesia	Male Wistar Rats	250 g	ICV administration •AgRP (0.05, 0.1, and 0.2 μg) Intranasal administration •HS014 (0 or 50 μg/10 μL)	•AgRP increased thermal thresholds in alcohol dependent animals, but had no effect on non-dependent nor alcohol naïve animals •ICV and intranasal administration of HS014 reverses alcohol-dependent hyperalgesia
Avegno et al. (21)	Alcohol-induced hyperalgesia	Male Wistar rats	300 g	Intra-CeA injection •HS014 (0.05, 0.1, or 0.2 ng) •α-MSH (0.3 or 1 μg) Intra-PAG injection •DAMGO (0.1 or 0.5 μg)	•HS014 decreased in alcohol-dependent rats whereas α-MSH increased in alcohol-naïve rats hindpaw withdrawal latency •Pre-treatment with DAMGO blocked α-MSH (CeA; 0.3 μg) pro-nociceptive effects
Zhao et al. (73)	CCI	Male Sprague-Dawley rats	200–250 g	Intrathecal injections •HS014 (50 μg/kg) •HS014 + SP600125 (10 μg/kg) *Drugs were given from day 3 to day 14 after the CCI surgery	•HS014 alone or in combination with with JNK inhibitor SP600125 alleviated thermal and mechanical hypersensitivity relative to CCI
Starnowska-Sokół et al. (74)	CCI	Male Albino Swiss CD-1 IGS mice	30–35 g	Intrathecal injections •Novel opioid agonist, MC4R antagonist hybrids (UW1 [parent opioid compound], UW3, UW5, UW9, UW10) •SHU9119 (0.09–23.27 [naïve mice]; 0.9–9.3 [CCI] nmol) •Naltrindole Hydrochloride (Delta-opioid receptor antagonist) •Naloxone (opioid receptor antagonist) •THIQ (2.5 nmol; MC4R agonist) •MTII (1.5 nmol; MC agonist)	In Naïve Mice: •SHU9119 had no effect on tail-flick In CCI Mice: •Both UW1 (parent opioid compound) and SHU9119 alleviated CCI mechanical hypersensitivity, but effects diminished after 90 min •Novel Hybrid compounds had lower ED_50_'s (UW3 = 0.0002; UW10 0.003; vs. parent opioid compound UW1 = 0.16 or MC4R antagonist SHU9119 = 3.33) and had longer antinociceptive effect •Pretreatment with opioid antagonists (Naloxone or Naltrindole) or Melanocortin agonists (MTII or THIQ) significantly reduced and/or completely abolished the anti-nociceptive effects of hybrid compounds UW3 and UW5 •Hybrid compounds had a significant antinociceptive effect compared to mixtures of UW1 and SHU9119
Piotrowska et al. (75)	CCI	Male Wistar rats	200–260 g	Intrathecal Admin •α-MSH (0.1, 10, 50 μg/5 μL) •ACTH (1, 10, 50 μg/5 μL) •CLIP (10 and 50 μg/5 μL •SHU9119 (1 μg/5 μL) *UW1 and UW5—novel bifunctional compounds with enkephalin analog and MC4R antagonist (0.001, 0.01, and 0.1 μg/5 μL)	•ACTH, α-MSH, and CLIP increased mechanical and thermal hypersensitivity after CCI. •α-MSH dose-dependently increased mechanical and thermal hypersensitivity •γ-MSH (1 μg/5 μL) decreased hypersensitivity, whereas (10 μg/5 μL) increased hypersensitivity •SHU9119 produced mechanical and thermal antinociceptive effects in CCI animals •UW3 and UW5 showed analgesic effects of all doses tested and lasted 2 h •In the management of neuropathy, UW3 and UW5 (0.1 μg/0.5 μL) exhibited longer analgesic efficacy relative to morphine in CCI animals treated for 9 days (starting on after day 7 of CCI)
Robinson et al. (76)	Red-haired male mice	MC1R^e/e^ male mice	8 weeks	PAG administration •Melanotan II (150 ng/2 μL) •Naloxone (3 μg/2 μL) •Naloxonazine (1.5 μg/2 μL)	PAG administration resulted in: •Decreased thermal nociceptive thresholds with MC4R agonism •Blockade of opioid receptors reduced nociceptive threshold
Klawonn et al. (77)	MC4R knockout mice	Male MC4R-STOP-flox	6-20 weeks old	Conditioning paradigm:•LPS (lipolysaccharide; 10 μg/kg) •HS014 (50 μg/5 μL	Conditioning results; •LPS injected mice displayed aversion to injection, MC4R lacking mice displayed preference to LPS and other aversive stimuli (lithium chloride, k-opioid receptor agonist) •WT mice treated with intranasal HS014 also displayed preference to LPS, greater time spent in hot plate, and antinociceptive effects

Other work indicating alternative melanocortin receptors included evidence that α-MSH could induce hyperalgesia in naïve rats (60) prior to MC4R cloning that occurred in the 1990's (26). The role of the melanocortin system in regulating pain-related behaviors was originally hypothesized based on interactions between melanocortins and opioids. POMC is an opioid prohormone that incorporates both melanocortin agonists (α-MSH and ACTH), and also opioid-receptor agonists (β-endorphin) into a core sequence (84). Opioids in general remain the gold-standard for managing acute pain, post-surgical pain, and pain related to cancer in humans, however the continued use of opioid analgesics in patient populations has reported that nearly a quarter of patients will discontinue medication use due to adverse side effects (85). One particularly problematic side effect is the induction of hyperalgesia following chronic opioid therapy, which has been reported in both populations of individuals suffering from pain conditions such as back pain and healthy volunteers (86). Studies indicated that melanocortin agonists such as α-MSH and ACTH antagonized the analgesic effects of morphine (79, 80, 87, 88). The melanocortin system and MC4R specifically are sensitive to effects of chronic opioid drug exposure. Administration of morphine in rats alters MC4R expression in a time- and region-specific manner, where prolonged administration leads to sustained reductions in MC4R expression in the periaqueductal gray (PAG), but a reversible decrease in the straitum (36). Furthermore, MC4R mRNA expression is decreased, but protein expression is increased in the dorsal root ganglion of morphine-treated male Wistar rats (68). Discrepancies in central and peripheral regions likely reflects region-specific neuroadaptations responding to enhanced opioid tone. For instance, a more time-specific investigation into opioid effects on MC4R expression was performed by Starowicz and colleagues. They found that acute administration of morphine decreases MC4R mRNA expression in the central nucleus of the amygdala (CeA) in male Wistar rats, whereas prolonged treatment with morphine significantly increased mRNA expression (47). The authors note however, that alterations in mRNA expression does not necessarily predict similar changes in protein expression (47). These changes highlight that MC4R regulation in response to morphine likely occurs in multiple directions depending upon where in the neural axis effects are studied.

### Melanocortin-Opioid Interaction Effects on Pain-Like Behaviors

Pharmacological antagonists that target both MC3R and MC4R (SHU9119, HS014) and those that have a selective affinity for MC4R (JKC-363) when given centrally to rats prevents tolerance and morphine-induced hyperalgesia (68, 70). Interestingly, subtherapeutic dosages of an MC4R antagonist, which has no effect on nociception in naïve animals, significantly attenuated tolerance to morphine (70). These results suggest an additive effect of MC4R blockade and opioid receptor agonism (68, 70), which has been further explored by bifunctional hybrid compounds. Recently, compounds that have a pharmacophore containing an enkephalin analog (Tyr-D-Ala-Gly-Phe) and MC4R antagonist derived from SHU9119 (Nle-c[Asp-His-2'-*D*Nal-Arg-Trp-Lys]) connected by various linkers have been developed. Results from these studies have indicated that these compounds have much lower effective doses as indicated by 50% effective dosages that were on the order of 1,000–10,000 times lower than parent compounds, for producing analgesia under preclinical models of nerve injury (i.e., chronic constrictive injury to the sciatic nerve) (74, 75). For instance, one of the hybrids (UW3) induced analgesia 1,500 times greater relative to the parent opioid receptor compound alone and 16,000 times greater to the parent melanocortin receptor compound alone, respectively in male CD-1 mice subjected to nerve injury (74, 75). Furthermore, these bifunctional compounds produced long lasting effects relative to the parent opioid compound or the MC4R antagonist compound throughout the testing period (i.e., they induced a significant mechanical and thermal anti-hypersensitive effect in neuropathic injured mice relative to either parent compound alone or a mixture of the parent compounds). Finally, pretreatment with MC4R agonists abolished the anti-hypersensitive effect of these hybrid compounds in nerve injured mice (74). These results support the notion that MC4R and opioid systems interact to influence pain-like responses and furthermore, due to the molecular nature of the linkers, these data support opioid-MC4R colocalization and/or dimerization *in vivo*. Mu-opioid receptors and MC4R co-localization has been identified in pain-processing regions such as the PAG, where nearly 50–70% of MC4R-positive cells contain μ-opioid receptors (89). It is likely therefore, that colocalization of these receptors in nodes along the pain-processing pathway mediate these effects, as recent evidence discussed below suggest a more central, rather than peripheral influence of MC4R (76) on pain processing.

### Melanocortin Effects in Other Pain Models

Beyond effects of enhancing morphine's efficacy and reduction in side effects, MC4R mediates alcohols negative effects including hypersensitivity. The link between alcohol abuse and hypersensitivity has been established where individuals either going through withdrawal from alcohol or those who have imbibed chronically exhibit hypersensitivity (4, 7), and importantly this is also seen in young adults (9) as well as individuals who abuse alcohol during adolescence (8). In preclinical models when animals exposed to chronic alcohol undergo withdrawal, hypersensitivity emerges during this period to thermal stimuli, which can be ameliorated with intranasal, intracerebroventricular (ICV), and site-specific CeA administration of the MC4R antagonist HS014 (20, 21). Furthermore, μ-opioid receptor activation in the ventrolateral PAG abolishes the pro-nociceptive effect of MC4R activation in the CeA; however, MC4R antagonism does not alter synaptic transmission in CeA cells projecting to the PAG (21). Thus, MC4R-related modulation of pain transmission may be due to MC4R effects on interneuron populations (90), reciprocal connections within the CeA (91), or projections from other regions such as the prefrontal cortex (92) that mediate pain processing.

MC4R also mediates pain-related behaviors in animal neuropathic (61, 65–67) and inflammatory pain models (62, 64, 77). Neuropathic pain remains a complex condition to treat, as pain relief is poorly managed in patients with less than half achieving effective pain relief (93). Preclinical models of neuropathic pain suggest a dysregulation of the melanocortin system in the dorsal root ganglion (DRG) and dorsal horn of the spinal cord. MC4R gene and protein expression has shown divergent changes following neuropathy, reportedly with protein expression increased (69) and gene expression either being decreased (48) or unchanged (61) in the DRG. This may be an adaptive mechanism wherein increased expression results in downregulation of MC4R at the transcript level. However, local intra-plantar injection of MC4R antagonists JKC-363 and SHU9119 ameliorate thermal and mechanical hypersensitivity to neuropathic pain, indicating that the increase in expression at the protein level promotes pain (69). The results of neuropathic pain on MC4R levels in other regions of the CNS are more consistent, with groups showing increases in MC4R in spinal regions, including the dorsal horn of the spinal cord, specifically in localized to layers I and II (65, 69, 94, 95), and supraspinal regions that mediate pain transmission including the PAG (72). Interestingly, MC4R antagonism not only reverses neuropathic-like pain states in preclinical models (48, 65, 69, 74, 75, 94), but these effects may be in part mediated via mitogen activated protein kinase (MAPK) pathways. In both DRG and the spinal cord, MC4R blockade decreased both MC4R cell numbers and p38 MAPK expression (71, 95). In addition, HS014 also significantly decreases JNK pathway activation and pro-inflammatory markers that are postulated to heighten neuropathic pain (73). Collectively, these data suggest that MC4R is, and remains, a highly important therapeutic target as MC4R antagonism attenuates pain-like behavior in multiple animal models. It is interesting to consider that antagonism of this receptor leads to anti-hyperalgesia, where discussed in the next section, agonism of the receptor alters the response to alcohol.

## Melanocortin System and Alcohol

### Effects of Alcohol on the Melanocortin System

Considerable work has been done on investigating the effects of alcohol on melanocortin receptors, its ligands, precursor products, and components of the melanocortin system. In response to *chronic* ethanol, POMC in hypothalamic sites has been reported to be increased (96), decreased (97–100), or not changed (101), although discrepancies in previous reports are likely due to methodological differences in administration of ethanol. For instance, De Waele and Gianoulakis (96) reported increases in β-endorphin in the arcuate nucleus and septum, however this was following 21 days of access to 10% volume/volume ethanol, although that study did not state when following the treatment animals were sacrificed for tissue processing (i.e., during withdrawal when alcohol levels in the blood are negligible, or if there was still alcohol in the system of the animals). Later studies indicated a time-dependent effect of alcohol on POMC precursors where, during alcohol treatment, animals display lower levels of POMC mRNA but following treatment there were increases in POMC mRNA (97, 98). Thus, the effect of alcohol and alcohol withdrawal likely affect POMC expression as has been reported with other systems affected by alcohol (24).

The endogenous agonist of MC4R, α-MSH, has been reported to be both decreased (102, 103) and increased (104, 105) following *chronic* ethanol exposure in rats. On the other hand, *acute* ethanol exposure produces decreases in α-MSH (102, 105, 106), POMC, and enzymes that cleave POMC to produce α-MSH including PC1 and PC3 (107). In *acute* alcohol exposure models, decreases in α-MSH fibers have been reported in the extended amygdala (bed nucleus of the stria terminalis; BNST), CeA, dorsomedial hypothalamus, paraventricular nucleus of the hypothalamus (102), and both fibers and cell bodies of the arcuate nucleus, a major hub that synthesizes the α-MSH precursor POMC (29, 107). Interestingly, there is also a decrease in the PC1 and PC3 enzymes within the arcuate nucleus as well (107). Data however is less clear following *chronic* ethanol exposure. Rainero et al. (103) as well as Navarro et al. (102) both describe decreases of either α-MSH or the precursor POMC within the arcuate nucleus, decreases of α-MSH in the BNST, CeA, lateral hypothalamus, pituitary, and substantia nigra after chronic alcohol exposure. This is contrasted to data from Kokare et al. (105) that showed increases in α-MSH in the CeA, and in hypothalamic divisions including the paraventricular nucleus, arcuate nucleus, and dorsomedial nucleus. Unfortunately, these data are difficult to reconcile as all studies used male Sprague-Dawley rats, liquid ethanol diets, and similar exposure timeframes; it is likely that alcohol effects are brain region- and strain-specific. For instance, 129/SvJ mice that exhibit decreased preference and consumption of alcohol also exhibit higher α-MSH fiber quantity in the medial amygdala, and conversely, lower fiber quantity in the lateral and dorsomedial hypothalamus, whereas C57BL/6 mice that exhibited higher preference for alcohol had an inverse expression pattern, with greater α-MSH fiber quantity in hypothalamic regions, but decreased fiber quantity in the medial amygdala (102).

Data from Kokare and colleagues shed light on what may occur during withdrawal: they reported an increase in α-MSH fiber immunoreactivity in animals treated with *chronic* ethanol exposure in regions including the arcuate nucleus and CeA (105). Supporting data has been seen with POMC mRNA expression that is potentiated weeks after chronic ethanol treatment has ended (98). Furthermore, MC4R expression is decreased, but α-MSH expression trends toward an increase within the CeA during withdrawal (21). The data thus far indicates that the melanocortin system responds both to acute and chronic alcohol challenges, however future studies are necessary to clarify acute and chronic ethanol effects on MC4R and its ligands over time.

### MC4R Effects on Alcohol Intake

MC4R agonism reduces ethanol intake [see (106) and Table 2]. This contrasts evidence presented above where MC4R antagonism reduces pain-like behaviors in multiple preclinical models. Initial evidence for the effects of MC4R signaling on alcohol intake was established by Ploj and collegues where in alko-accepting rats (AA) bred for high ethanol preference, MTII, a non-selective MC3R and MC4R agonist, reduced ethanol consumption when given via the ICV route (108). Importantly, MC4R antagonism with HS014 had no effect on alcohol intake indicating that pharmacological agonism, but not antagonism altered ethanol intake (108). This has been consistently affirmed in both rat and mouse models of alcohol drinking across different paradigms (Table 2). Furthermore, broad and site-specific administration of MC4R agonism has delineated specific neural pathways involved in modulation of both ethanol intake and characteristics of ethanol including palatability.

**Table 2:** Melanocortin system interactions on alcohol drinking.

**References**	**Animal model**	**Age and/or Weight**	**Drinking paradigm**	**Drugs**	**Outcomes on drinking behavior**
Ploj et al. (108)	Female AA rats bred for alcohol consumption	116–177 g at beginning and 141–213 g at the end	Rats were given two bottle choice after 7 days of only ethanol drinking (10% v/v) throughout the experiment	ICV administration •HS014 (1nmol/rat) •MTII (1nmol/rat)	•MTII significantly reduced consumption and preference for ethanol, which returned to baseline values •HS014 had no effect on drinking
Navarro et al. (109)	C57BL/6NTac (mouse line bred to prefer alcohol)	25–30 g	Two bottle choice (8% w/v EtOH) for 2 weeks then 8 h sessions of ethanol, food and water intake	ICV administration •AgRP 83–132 (5.0 μg) •MTII (1.0 μg) Intraperitoneal injection •MTII (100 or 150 μg)	Central Administration: •MTII significantly decreased EtOH and food intake. Effect was blocked by pretreatment with AgRP Peripheral Administration •150 μg MTII decreased EtOH and food consumption, 100 μg MTII decreased food consumption
Navarro et al. (110)	MC3R deficient (Mc3r^−/−^) MC3R mice (Mc3r^+/+^) on a C57BL/6J background	8–12 week mice	Two bottle choice (20% v/v EtOH)	ICV administration •AgRP 83–132 (0.05 or 0.1 μg) •MTII (1.0 μg) •*Selective MC4R agonist: cyclo*(NH-CH_2_-CH_2_-CO-His-d-Phe-Arg-Trp-Glu)-NH_2_ (1.0 or 3.0 μg) Intraperitoneal injection •MTII (10 mg/ka)	Central Administration •MTII reduced Ethanol drinking and preference in both Mc3r^−/−^ and Mc3r^+/+^ mice •Selective MC4R agonist dose-dependently decreased ethanol and food intake •AgRP increased ethanol intake only at the 0.05 μg dose Peripheral Administration •MTII reduced Ethanol drinking and preference in both Mc3r^−/−^ and Mc3r^+/+^ mice •MTII also decreased food intake in both strains
Polidori et al. (111)	Marchig-Sardinian	360–380 g	2 and 24 h access •10%w/v	ICV (LV and third ventricle; 3 V) Administration •MT (0.1 [only dose used for 3 V]and 1 nmol) •SHU9119 (0.5 nmol) •AgRP (1 nmol)	Central Administration •MTII reduced Ethanol drinking only at a dose of 1 nmol in the LV as a single dose •Repeated administration of MTII over 5 days in animals with 24 h access to EtOH during the first 8 and 24 h, but not the first 2 h •Antagonism had no effect on alcohol intake
Navarro et al. (112)	AgRP deficient (AgRP^−/−^) mice Wildtype mice (AgRP^+/+^) on C57BL/6J background	8 weeksMale and Female for Two-bottle choice and Bing-like ethanol drinking	•Operant conditioning (8% v/v EtOH; 1–3% sucrose, 0.01–0.1% w/v saccharin) •Two-bottle choice (8% v/v EtOH) •Binge-like EtOH •(20% EtOH) •EtOH-induced sedation (4.0 g/kg; 19% w/v intraperitoneal injection)	None	•AgRP^−/−^ mice exhibited significantly less ethanol lever pressing and consumption ° Similar effect in binge condition; AgRP^−/−^ mice exhibited reduced ethanol drinking and lower blood alcohol levels •Both male and female AgRP^−/−^ mice exhibited less preference for ethanol in the two-bottle choice •AgRP likely positively modulates ethanol drinking
Navarro et al. (113)	MC4R deficient mice (MC4R^−/−^) MC4R wildtype (MC4R^+/+^) on a C57BL/6J background	6 week	•Two-bottle choice •(3–20% v/v EtOH)	ICV administration •MTII (0.5 or 1.0 μg) Intraperitoneal injection •MTII (5 mg/kg)	Two-bottle choice •Both geneotypes chose to escalate ethanol at higher percentages and drank saccharine in similar amounts •MC4R^+/+^ mice drank more water than MC4R^−/−^ Central Administration •MTII significantly decreased ethanol drinking and food intake in MC4R^+/+^, but not MC4R^−/−^ •In wildtype mice MTII (0.5 μg) reduced food, sucrose, and saccharine but no affect on water consumption Peripheral administration •MTII reduced food and ethanol intake regardless of genotype
York et al. (114)	Male Alcohol preferring (P) and non-preferring (NP) rats	6 weeks(first cohort; P: 299 g, NP: 269; second cohort; P: 284, NP: 310)	•Two bottle choice •(7.5% w/v)	Intra-CeA injections •MTII (0.5 nmol) •SHU9119 (1 nmol) •HS104 (1 nmol)	CeA Administrations: •In P rats, MTII suppressed alcohol intake 12 h after injection and also 24 h after injection •SHU9119 also suppressed alcohol intake only after 24 h in P rats •HS014 increased food intake and decreased preference for alcohol in P rats only at 24 h post-injection, but had no effect on alcohol consumption
Lerma-Cabrera et al. (115)	Male Sprague Dawley rats	280–300 g	Two-bottle choice (6% w/v) for a month	VTA, NAc, or LH injections •HS014 (0.02 or 0.05 μg) •*cyclo*(NH-CH_2_-CH_2_-CO-His-d-Phe-Arg-Trp-Glu)-NH_2_ (0.75, 1.5 μg)	Injections into the VTA and NAc •MC4R agonism reduced the amount of voluntary ethanol •In the NAc, 0.2 μg of HS014 increased ethanol consumption relative to the 0.05 μg dose but not relative to saline
Lerma-Cabrera et al. (116)	Male Sprague Dawley rats	280–300 g	Two-bottle choice (6% w/v) for a month and then taste reactivity testing	NAc or LH injections •*cyclo*(NH-CH_2_-CH_2_-CO-His-d-Phe-Arg-Trp-Glu)-NH_2_ (0.75, 1.5 μg)	NAc but not LH injections showed: •decrease in the hedonic reactions to ethanol for both concentrations •increase in aversive responses to ethanol for the 0.75 μg but not the 1.5 μg dose
Olney et al. (117)	Male and Female MC3R^−/−^ and MC3R^+/+^	Not mentioned	DID paradigm (20% EtOH)	ICV administration •MTII (0.25, 0.5, or 1.0 μg)	DID induced: •High levels of EtOH consumption •Following the first hour of DID MC3R^−/−^ mice were more sensitive to MTII showing blunted ethanol intake at all doses tested whereas MC3R^+/+^ mice only showed blunted EtOH intake at 1.0 μg •At 4 h only the MTII was effective in both genotypes at 1.0 μg
Navarro et al. (118)	Male C57BL/6J mice	20–25 g	Drinking in the Dark (20% v/v)	Intraperitoneal injections •MTII (0.3, 1, 3, 10 mg/kg) •Naltrexone (0.3, 1, 3, 10 mg/kg) Combination injections •MTII (0.3, 1, 3 mg/kg) + Naltrexone (0.82 or 1.64 mg/kg) •Naltrexone (0.3, 1, or 3 mg/kg) + MTII (0.26 or 0.52 mg/kg)	DID ethanol intake •Naltrexone (3 and 10 mg/kg) and MTII (dose 1, 3, 10 mg/kg) significantly decreased ethanol consumption and blood alcohol levels
Carvajal et al. (119)	Male Sprague Dawley rats	PND 25 when experiments began	Binge Ethanol Protocol (intraperitoneal injections of 25%w/v EtOH; 2 injections every 4 days for 2 weeks) Intermittent ethanol access (20% v/v; 3 x per week for 45 days)	NAc Shell injections •*cyclo*(NH-CH_2_-CH_2_-CO-His-d-Phe-Arg-Trp-Glu)-NH_2_ (0.75, 1.5 μg)	•MC4R antagonism reduced binge-like ethanol consumption
Sprow et al. (120)	Male C57BL6/J	6–8 weeks and 23 g	Drinking in the Dark (DID) cycle (20% v/v EtOH)	LH, DS, BNST infusions •MTII (0.5 nmol) •AgRP (0.1 nmol) BNST infusion •MTII (0.5 nmol) •AgRP (0.1 nmol)	LH injections: •MTII decreased ethanol consumption in the first hour but not second of DID •AgRP increased ethanol consumption but was not changed in the LH following DID (separate experiment) BNST injections •MTII significantly decreased ethanol drinking, whereas AgRP had no effect DS injections had no effect on alcohol drinking
Zhou et al. (121)	Male and Female C57BL/6 Male nPE^−/−^ mice (knockout of POMC enhancers)	9–10 weeks of agenPE^−/−^ mice weighed 40 g and 35 g (m, f) and nPE^+/+^ mice weighed 27 and 23 g (m,f)	2 main arms 1. Drinking in the dark (DID) with 15% v/v EtOH for 4 days 2. Chronic intermittent access w/two bottle choice with either single injection or 4 injections on the last 4 days of testing (one injection each day)	Drugs (given intraperitoneally):DID experiment •Buproprion: 5, 10, 20 mg/kg •Naltrexone: 1 or 2 mg/kg •Combination (Buproprion + Naltrexone): 5+0.5, 10+1, 20+1, 20+2 mg/kg •HS015 (0.5 μmol/kg) Intermittent access •Combination (10+1 mg/kg)	DID experiment •Buproprion had no affect on drinking alone •Naltrexone decreased drinking at 2 mg/kg in both sexes •Combination of 10+1 Buproprion and Naloxone decreased drinking greater in males than females ° Also decreased sucrose drinking in males but not females ° No effect on non-caloric reinforcer saccharin ° HS014 blocked the combination of buproprion and naloxone on drinking behaviors •With nPE^−/−^ mice the combination of 10+1 Bupropion and Naltrexone had no effect on drinking Intermittent access •Combination treatment reduced drinking in males but not females •No tolerance to multiple administration of drug

The role of MC4R in regulating ethanol drinking was confirmed by a study from Navarro and colleagues where they showed that MTII when given ICV reduced ethanol drinking in MC4R^+/+^ mice, but not MC4R^−/−^ mice (113). Furthermore, they saw that this effect was not only limited to ethanol intake but also other caloric sources including food and sucrose and non-caloric reinforcers such as saccharin (113). Multiple studies have confirmed that agonism of MC4Rs blunts ethanol intake in diverse alcohol intake paradigms including voluntary consumption (108–111, 114, 115), and binge-like drinking (112, 117–121) in both mice and rats. Studies have indicated that the reduction in ethanol intake is associated with decreases in caloric intake as food was decreased following agonism of MC4R (109, 114, 119). Although it is well-known that MC4Rs mediate food intake (29), and that agonism of MC4R decreases feeding (122, 123) the effects on ethanol intake are likely dependent on non-homeostatic mechanisms of caloric regulation. For example, York and colleagues described a decrease in ethanol intake in alcohol preferring rats when MTII was given into the CeA and basolateral amygdala (BLA) regions that lasted longer than observed decreases in food intake (114). Furthermore, when rats were calorically controlled (i.e., animals receiving drug had their calorie intake matched to control animals to investigate changes in alcohol consumption), effects of MC4R agonism resulted in prolonged depression of ethanol intake and preference (114). These data indicate that, at least in certain nodes of the neuraxis, MC4R modulation of ethanol intake depends on homeostatic and non-homeostatic mechanisms. In support of non-homeostatic related-mechanisms of MC4R modulation on ethanol intake, targeting MC4Rs located in the arcuate nucleus, a key node in the regulation of homeostatic feeding control (26, 29), agonism had no effect on ethanol intake in alcohol-preferring rats (111).

MC4R antagonism has fewer clear effects on ethanol intake in preclinical models. It has been reported that MC4R antagonism has no effect (108, 109), a decreased effect (114), and an increased effect (110) on ethanol intake. Discrepancies in these findings may be due to (1) location of pharmacological agents administered into the CNS, (2) temporal effects of pharmacological agents, (3) duration of ethanol exposures, (4) pharmacological agents that were used, and (5) species. Navarro and collegues found that a low (0.05 μg) but not high (0.1 μg) dose of AgRP increased ethanol intake in the two bottle choice model in C57BL/6J mice (110). This is in contrast to data from Ploj and colleagues that showed no effect of the selective antagonist HS014 on ethanol intake in female alcohol-preferring rats (108), and is also in disagreement from an earlier study by the same group showing that higher doses of AgRP (5.0 μg) had no effect on ethanol intake (109). It may be that lower doses of AgRP effectively increase ethanol drinking, whereas higher doses have no effect. In line with the literature, pretreatment with either AgRP (110) or HS014 (121) effectively blunts MC4R agonist-induced decreases in ethanol drinking. A decrease in ethanol drinking following administration of SHU9119, an MC4R antagonist, has also been reported in male rats that prefer alcohol (114). However, the decrease in ethanol drinking was concluded to be likely due to secondary effects on water consumption, which increased in both the amount and preference, prior to the decrease in ethanol intake (114). Finally, this decrease in ethanol intake was replicated with intra-amygdalar injections (114), thus the discrepancies may be due to site-specific modulation of MC4Rs. For instance, administration of MTII into the NAc or VTA reduces the amount of voluntary ethanol intake in rats (115), whereas administration into the lateral hypothalamus or the third ventricle (111) has no effect on ethanol intake. In other addiction-related brain regions, such as the nucleus accumbens shell, MC4R agonism results in a decrease in the hedonic effects and an increase in the aversive effects of ethanol (115). Importantly, the effects of MC modulation are limited to ethanol intake, and do not extend to consummatory behaviors. Injection of MTII into the posterior VTA, a region important for alcohol reinforcement, had no effect on operant conditioning responses alone (i.e., lever presses in the absence of a reinforcer were not affected) (124). Taken together, the decrease in ethanol intake seen following MC4R agonism is likely the result of a combination of non-hedonic factors that include increases in ethanol aversion and homeostatic behaviors including a decrease in the drive to consume caloric compounds.

### Opioids and Melanocortin Interactions Effects on Alcohol Intake

MC4R agonism reduces the hedonic value of alcohol (110) while increasing the aversive effects of ethanol (119). It is well-known that opioids also influence the intake and hedonic nature of ethanol. In general, opioid agonism increases ethanol intake whereas antagonism decreases ethanol intake [for a review see (125)]. Blockade of opioid receptors not only modulates ethanol intake but also the hedonic effects of alcohol, similar to MC4R. Opioid receptor antagonism with naltrexone increases aversive responses to ethanol and shifts the palatability of ethanol in rats at low (126) and high doses (127). Naltrexone is FDA approved for alcohol addiction, whereas bupropion is a dopamine and norepinephrine reuptake inhibitor that is approved for depression and smoking cessation (128). To our knowledge, bupropion does not activate MC4R directly however there is evidence supporting an indirect activation of MC4R via modulation of POMC and/or α-MSH expression (106, 129). In animals treated with a high-fructose corn syrup diet, decreases in hypothalamic POMC expression were abrogated by bupropion and furthermore, bupropion also increases expression of BDNF in the hippocampus in rats (129). Importantly, BDNF can bind to the promotor region of POMC, thereby enhancing expression, and thus driving melanocortin tone (130). Electrophysiological data suggests that bupropion enhances POMC-neuronal firing within the arcuate nucleus, likely though a dopamine-dependent mechanism (131). Therefore, bupropion may enhance melanocortin tone either through increases in POMC expression and/or activation of MC4Rs indirectly via increased α-MSH release at terminal sites.

Pharmacological manipulation of the opioid system with bupropion supports the idea of opioid-melanocortin interaction effects on drinking behaviors. Bupropion when given at subtherapeutic dosages in conjunction with naltrexone (10 and 1 mg/kg, respectively; intraperitoneal injection) reduced ethanol intake only in male mice, but not female mice (121). This effect was not seen in mice that were lacking nPE, a promotor essential for POMC production. Blockade of MC4R with HS014 blunted the combined effects of naltrexone and bupropion on alcohol intake, indicating that bupropion and naltrexone-induced decreases in ethanol drinking are dependent on MC4R (121). Further implicating an interaction between MC4R and opioid systems on alcohol drinking, Navarro and colleagues reported that a subtherapeutic dose of MTII increased the effectiveness of naltrexone by 7.6-fold in blunting ethanol intake (118). It should be reiterated that although there is a functional interaction between the opioidergic and melanocortin systems in effects on both alcohol and pain processing, they are dichotomous. Specifically, MC4R antagonism in conjunction with opioid-receptor agonism reduces pain-like behaviors, whereas MC4R agonism and opioid-receptor antagonism seem to decrease ethanol intake in preclinical models.

## Neurobiology of Alcohol-Induced Changes in Pain Processing

### Bi-Directional Interactions Between Pain and Alcohol

As mentioned above, hyperalgesia can occur following chronic alcohol exposure in humans. Males undergoing alcohol withdrawal exhibit increased sensitivity to thermal pain in peripheral sites including the hand and sternum (7). In a large study with nearly 8,000 patients that had substance use disorder, nearly 62% reported chronic pain, and of those who reported moderate to severe pain, nearly half used alcohol heavily (132). Furthermore, following serious injury, prior alcohol use was associated with persistent chronic pain outcomes following injury (17, 133). Importantly, neither current alcohol use (17) nor past pain intensity (133) seemed to have an effect on chronic pain a year or more following a traumatic event. This evidence likely indicates that pre-injury alcohol exposure sensitizes an individual toward a chronic pain state following a traumatic and/or stressful injury. Finally, there is evidence supporting the idea that in humans, early exposure to alcohol increases the risk for maladaptive outcomes. The National Epidemiologic Survey on Alcohol and Related Conditions (NESARC) reported that drinking prior to age 14 increased the likelihood of subsequent alcohol dependence by about 50% (134). Although the data is limited, some studies have identified changes in pain-related outcomes following alcohol drinking in young adults. In a study with 417 adolescents being treated for alcohol and substance abuse, findings showed a significantly greater incidence of pain-related disorders in those being treated for alcohol and substance abuse than those without these diagnoses (8). Specifically, there was a higher prevalence for headaches and gastrointestinal pain, as well as reproductive systems pain (i.e., endometriosis) in females (8). In college-aged students, hypersensitivity to pressure emerges in binge drinkers who have consumed alcohol within the past 2 days relative to binge drinkers who have not consumed alcohol, and also relative to non-binge drinkers (9). Some human studies have also investigated longitudinal effects of alcohol use on pain-related outcomes. In a 3-year study, 401 individuals were followed, assessed for either problematic or non-problematic drinking levels and monitored for pain levels. In both men and women, problematic drinkers were more likely to use alcohol to manage their pain, and those with more pain-like symptoms drank more than those with less pain-like symptoms (135). This study highlights that there is a bi-directional relationship between pain and alcohol use. Collectively, the above data indicate that alcohol may augment maladaptive responses to insults, and this likely occurs in both adults and adolescent drinkers. There is also an abundance of preclinical evidence for alcohol effects on pain-related brain systems, and for the development of alcohol-induced hyperalgesia. It seems that both clinically and preclinically, when in the system, alcohol leads to analgesia (4), whereas during withdrawal when no alcohol is in the system, this produces hyperalgesia (19). Neural regions that are sensitive to alcohol and mediate nociceptive signaling are likely therapeutically relevant areas to investigate these mechanisms.

### A Role for MC4R Signaling in Pain-Alcohol Interactions

As outlined above, MC4Rs likely mediate diverse effects on pain and alcohol-related behaviors via dichotomous effects at the receptor level. Specifically, antagonism of MC4R seems to reduce pain-related behaviors in preclinical models, whereas agonism reduces ethanol intake in preclinical models. A key question that has yet to be elucidated are what molecular mechanisms at the MC4R level are occurring to mediate these divergent behaviors? MC4Rs are coupled to all three major classes of G proteins including G_s_, G_i/o_, and G_q_ that modulate secondary messengers including cAMP, phospholipase C-dependent signaling cascades, and the mitogen activated kinase pathways (MAPK) (83). Furthermore, MC4R couples to the inhibitory g-coupled protein G_i/o_, where activation stimulated pertussis toxin sensitive GTPγS binding, that was also stimulated by AgRP (136). Downstream of these signaling events, MC4R-induced activation leads to MAPK activation (137, 138), AMP-kinase activation (139), and c-jun kinase activation (140). These pathways are critical for regulating substates of excitatory transmission, which are likely increased during withdrawal from alcohol, a period that is characterized by hyperalgesia (4, 18, 19). MC4R activation increases intracellular calcium via increased cAMP production and protein kinase A activation (141). This activation leads to an upregulation of AMPA receptors via a G_s_-PKA-dependent mechanism (142, 143). Furthermore, MC4R activation via this G_s_ pathway increases excitatory transmission in pain-related brain regions including the parabrachial nucleus (42). As melanocortin system activity is likely increased during the withdrawal period (4, 18, 19, 105), potentiated G_s_-PKA signaling likely contributes to hyperalgesia through potentiated excitatory transmission in pain-related regions.

In addition to the canonical G-coupled protein pathways, MC4R activates downstream mediators including MAPK extracellular-signal-related kinases (ERKs) 1 and 2 in a manner that is dependent on the cell expression system and ligand binding. In HEK293 cells, ERK1/2 activation was dependent on G_i/o_ activation, as the specific inhibitor pertussis toxin reduced MC4R-mediated ERK1/2 activation (144). This is contrasted to studies conducted in GT1-1 or GT1-7 cells, that found ERK1/2 activation was unaffected by pertussis toxin, however when MC4R was stably expressed in human HEK293 cells, pertussis toxin abrogated phosphorylated ERK1/2 activation (144). Importantly, the NDP-MSH (an agonist of MC4R) mediated activation of ERK1/2 in both HEK293 and GT1-1 cells was blocked by MC4R antagonist SHU9119 (136, 144). As protein kinase A and ERK activity is associated with chronic pain-like states in rats (71, 95, 145, 146), blockade of ERK activity following MC4R antagonist HS014 treatment is associated with decreases in pain-like responses (71). These effects may be directly mediated by blockade of MC4R with selective antagonists, or they may be mediating decreases in ERK activity through a potentially different mechanism as discussed below.

The endogenous antagonist AgRP can function both as a competitive antagonist inhibiting α-MSH binding to MC4R, and also serving as an inverse agonist, decreasing the amount of cAMP formed by MC4R (43, 147–149). *In vivo* data supports this notion as AGRP induces long-lasting effects on food intake beyond exposure to the drug (150). In addition to inhibition of G_s_ pathways and activation of G_i/o_ pathways, AgRP mediates endocytosis via β-arrestins (148). Specifically, binding of either α-MSH or AgRP induced β-arrestin-1- and 2-mediated endocytosis in HEK293 cells, and this effect was blunted in cells lacking the β-arrestin proteins (148). Importantly, this was a reversible process, where 1 h following treatment and removal of α-MSH or AgRP, receptors were relocated to the plasma membrane rather than degraded, and the β-arrestin effects were confirmed in a hypothalamic cell line (148). Further complicating the effects of AgRP, a more recent studied identified that AgRP activates ERK1/2 in a G_q_-dependent manner as phosphatidylinositol 3-kinase inhibitors decreased ERK1/2 activation following AgRP treatment in cultured GT1-7 hypothalamic cells (151). Like AgRP, α-MSH induces a β-arrestin-dependent internalization that occurs when agonist ligands toward MC4R come into contact with the receptor, and this internalization occurs on the timescale of minutes (152). This β-arrestin internalization was dependent on a protein kinase A, β-arrestin-1/clathrin, but not caveolae-pit mechanisms in HEK293 and COS-1 cells (152). A two-photon microscopy study utilized rhodamine labeled antagonist SHU09119 showed that in the presence of an antagonist, MC4R remained on the surface of HEK293 cells for >30 min, whereas, the agonist led to internalization within 10 min (153). These data indicate that antagonists likely reduce signaling through major MC4R-related pathways including G_s_-cAMP-PKA, and do not recruit β-arrestin complexes, likely preventing desensitization of MC4Rs. The effects of synthetic MC4R antagonists on G_i_ signaling remains to be determined and, more generally, more work is needed to determine how specific antagonists (and potentially biased ligands) modulate both G_s_- and G_i_-signaling pathways, and how chronic alcohol and other drugs of abuse affect signaling properties.

## Conclusions

*How does MC4R activation mediate reductions in alcohol drinking but increases in pain-like responses, while pain drives alcohol drinking and excessive alcohol use drives pain?*

One potential answer to this question is the interaction between MC4Rs and brain-derived neurotrophic factor (BDNF). MC4Rs are localized in neuronal cells (49, 154), astrocytes (155), and microglia (156). MC4R agonism results in BDNF production (157) and, although BDNF production is dependent on cre-Response Element Binding Protein (CREB) induction by PKA, recent studies have indicated that MC4R may generate BDNF through exchange factors (EPACs) via ERK1/2, and not via a PKA-dependent mechanism (158). Polymorphisms within the BDNF gene are correlated with susceptibility toward addictive behaviors, including alcohol abuse (159) and chronic pain phenotypes (160). Hypofunction of BDNF may be involved in the molecular processes that underlie excessive alcohol intake (161). In individuals withdrawing from alcohol, although BDNF levels are unchanged relative to control patients, they do correlate negatively with withdrawal symptoms (i.e., lower levels of BDNF are associated with greater withdrawal symptoms) (162). In alcohol preferring male P rats, BDNF expression was decreased in the CeA relative to non-alcohol preferring NP male rats (163). Furthermore, knockdown of BNDF expression in the CeA via antisense oligodeoxynucleotides CeA increased alcohol intake in male Sprague-Dawley rats in a two-bottle choice paradigm, and this effect was reversed by co-infusion of BDNF in the CeA (163). Administration of BDNF into the striatum, specifically the dorsolateral and dorsomedial striatum reduces ethanol intake, and conversely, decreases in BDNF mediated by short interfering RNA, increases ethanol intake (164). We hypothesize targeting MC4Rs that preferentially activate BDNF through a PKA-independent mechanism may ameliorate alcohol-related pathologies while also reducing excitatory transmission through a G_s_-PKA dependent pathways, leading to a reduction in pain-related behaviors.

**Figure 2:**
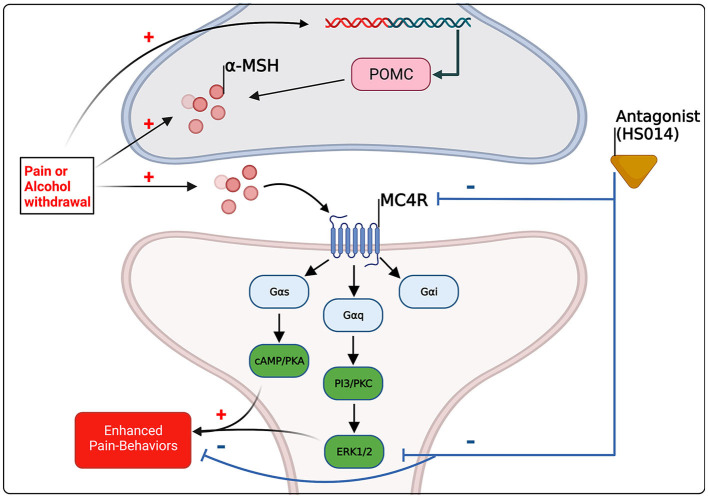
Schematic summary of the evidence-based and hypothetical effects of pain and alcohol withdrawal on melanocortin signaling within the central nervous system, and of the effects of altered melanocortin signaling on pain-related behaviors. Pre-clinical evidence suggests that pain and alcohol withdrawal [see (98, 105)] are associated with increased melanocortin tone through increases in α-MSH and/or POMC in brain regions implicated in pain and substance abuse, including the amygdala. MC4R couples to all three major G-coupled protein pathways, however activation of PKA and the mitogen activated protein kinase (MAPK) ERK1/2 are associated preclinically with pain-states (145, 146), and antagonism of MC4R reduces pain-related outcomes and results in decreases in ERK1/2 activation (72). The molecular mechanisms underlying MC4R drug effects on alcohol intake remain unclear.

In conclusion, the effects of alcohol and pain on MC4R are congruent; it seems that, preclinically, chronic pain increases melanocortin tone, and following chronic alcohol exposure during withdrawal, when hyperalgesia emerges, there is likely an increase in melanocortin tone (see Tables 1, 2). It should be noted however, studies focusing on chronic alcohol effects are limited and further studies are needed to resolve spatial and temporal changes. It is likely that upregulation of melanocortin tone either following pain conditions or during withdrawal promotes pain-like behaviors through G_s_-coupled pathways, for example, via PKA activation and subsequent MAPK activation including ERK1/2 (see Figure 2). What has yet to be resolved is the dichotomous nature of MC4R signaling, where antagonism reduces pain-like behaviors yet agonism decreases alcohol drinking. Again, as postulated by Koob and colleagues, during the withdrawal/negative affect stage of addiction, pro-stress neural systems such as CRF are upregulated and potentiated (53, 55). As melanocortin tone is increased following stressors (58, 59), it may be postulated that upregulated melanocortin tone during withdrawal promotes pain-like behaviors. Agonism of MC4R decreases ethanol consumption, beyond what would be expected from mechanisms of energy homeostasis (102, 112) (also see Table 2). Therefore, the increase in melanocortin tone following withdrawal may serve to blunt further excessive alcohol intake, and lead to restoration of homeostasis. It should also be noted that alterations in melanocortin tone are likely dependent on rodent species/strain and brain region, thus, further work is necessary to characterize alterations to the melanocortin system and MC4R to obtain a more holistic view of what occurs during withdrawal. Combinations of opioid and MC4 receptor bifunctional compounds (74, 75) may reveal opportunities for simultaneously antagonizing opioid receptors and initiating MC4R signaling that is biased toward either G_i/o_ or G_q_ pathways that promote BDNF production. More work is needed to fully characterize the effects of chronic alcohol exposure on the brain melanocortin system in adults and adolescents. Melanocortin-4 receptors modulate alcohol- and pain-related outcomes, as well as hyperalgesia observed after chronic alcohol exposure, and thus may hold promise as a novel therapeutic target for improving quality of life in individuals living with AUD or chronic pain or both of these disorders.

## Author Contributions

NS and NG conceptualized the manuscript and edited the manuscript. NS drafted the manuscript. Both authors contributed to the article and approved the submitted version.

## Funding

This work was supported by National Institutes of Health Grant F30 AA028691 to NS, R01 AA023305 to NG, and by a Merit Review Award from the United States Department of Veterans Affairs, Biomedical Laboratory Research and Development Service Grant I01 BX003451 to NG.

## Conflict of Interest

The authors declare that the research was conducted in the absence of any commercial or financial relationships that could be construed as a potential conflict of interest.

## Publisher's Note

All claims expressed in this article are solely those of the authors and do not necessarily represent those of their affiliated organizations, or those of the publisher, the editors and the reviewers. Any product that may be evaluated in this article, or claim that may be made by its manufacturer, is not guaranteed or endorsed by the publisher.
